# Physiological and transcriptomic analyses reveal tea plant (*Camellia sinensis L.*) adapts to extreme freezing stress during winter by regulating cell wall structure

**DOI:** 10.1186/s12864-023-09670-1

**Published:** 2023-09-20

**Authors:** Jinlei Luo, Shuangjie Huang, Yali Chang, Hui Li, Guiyi Guo

**Affiliations:** College of Tea Science, Henan Key Laboratory of Tea Plant Comprehensive Utilization in South Henan, Xinyang Agriculture and Forestry University, 46400 Xinyang, Henan PR China

**Keywords:** Tea plant, Natural freezing stress, Cell wall, Transcriptomic analysis

## Abstract

Tea plants grown in high-latitude areas are often damaged by extreme freezing temperatures in winter, leading to huge economic losses. Here, the physiological and gene expression characteristics of two tea cultivars (Xinyang No. 10 (XY10), a freezing-tolerant cultivar and Fudingdabaicha (FDDB), a freezing-sensitive cultivar) during overwintering in northern China were studied to better understand the regulation mechanisms of tea plants in response to natural freezing stress. Samples were collected at a chill (D1), freezing (D2) and recovery (D3) temperature in winter. TEM analysis of integrated leaf ultrastructure at D2 revealed lower malondialdehyde and relative electrical conductivity in XY10 than in FDDB, with serious cell structure damage in the latter, indicating XY10 was more resistant to freezing stress. Differential gene expression analysis among the different samples over winter time highlighted the following gene functions in cell wall metabolism (*CesAs*, *COBLs*, *XTHs*, *PGs*, *PMEs*), transcription factors (*ERF1B* and *MYC2*), and signal transduction (*CDPKs* and *CMLs*). The expression pattern of cellulose and pectin-related genes suggested higher accumulation of cellulosic and pectic materials in the cell wall of XY10, agreeing with the results of cell wall and its components. These results indicated that under the regulation of cell wall genes, the freezing-resistant tea cultivar can better maintain a well-knit cell wall structure with sufficient substances to survive natural freezing damage. This study demonstrated the crucial role of cell wall in tea plant resistance to natural freezing stress and provided important candidate genes for breeding of freezing-resistant tea cultivars.

**Supplementary Information:**

The online version contains supplementary material available at 10.1186/s12864-023-09670-1.

## Introduction

Tea plant (*Camellia sinensis (L.) Kuntze*) is an important commercial woody crop native to tropical and subtropical climates in southwest China. In the context of frequent global extreme climate events, low temperature freezing injury that occurs in winter and early spring often causes great damage to the growth of tea plants and reduces tea yield and quality, especially in tea producing areas at high latitudes and high altitudes [1]. Xinyang City is the largest green tea production area in Henan Province, China, where tea plants could be affected by different degrees of freezing damage almost every winter. This suggested that breeding and planting freezing-resistant tea cultivars and developing effective anti-freezing measures are important issues in local tea production. Tea plant “Xinyang No. 10” (XY10) is a national superior cultivar bred from local species in Xinyang, which possesses strong freezing resistance through long-term adaptation to low temperature is an ideal material for studying the antifreeze mechanisms in tea plants [2]. However, to our knowledge, no study has been performed on the antifreeze mechanism of XY10 so far.

In recent years, with the rapid development of high-throughput sequencing technology, transcriptomic technology has become an effective means to identify genes related to low temperature resistance of tea plants. For example, Wang et al. [3] performed the first RNA-Seq and digital gene expression (DGE) analysis on the leaves of tea plants during natural cold adaptation and de-adaptation process, and the key genes were identified to be mainly involved in calcium signal regulation, protein kinase, lipid metabolism and carbohydrate metabolism. Additionally, Wang et al. [4] observed the overwintering transcriptional changes of different cold-tolerant tea cultivars in a natural environment of southern China and demonstrated the involvement of the oxidative stress-activated reactive oxygen species (ROS) gene network in tea plant resistance to cold stress. Moreover, Li et al. [5] analyzed the differences in transcriptional changes between small- and large-leaf tea cultivars under controlled cold acclimation in an artificial chamber, and concluded that the stronger cold tolerance of small-leaf tea cultivar is related to the differential expression of genes involved in ABA signal conduction, photoinhibition, and plant immunity. However, most of the existing studies are focused on the cold adaptation process of tea plants to low temperature above zero, and little attention has been paid to how the freezing resistant tea cultivars adapt to the sub-zero temperatures in severe winters, although understanding this is important for the northern tea production regions with frequent extreme low-temperature events.

In this study, physiological and transcriptomic analyses were performed on XY10 leaves exposed to a natural sub-zero temperature during winter, using tea cultivar Fudingdabaicha (FDDB) planted in the same plot as the control. The results of this study facilitate the understanding of physiological and genetic regulatory changes involved in freezing resistance in different freezing-tolerant genotypes. Additionally, some key genes associated with regulation, signalling and cell wall biosynthesis were identified on the basis of comprehensive comparative analysis.

## Results

### Physiological changes of the two tea plant cultivars during natural wintering

**Fig. 1:**
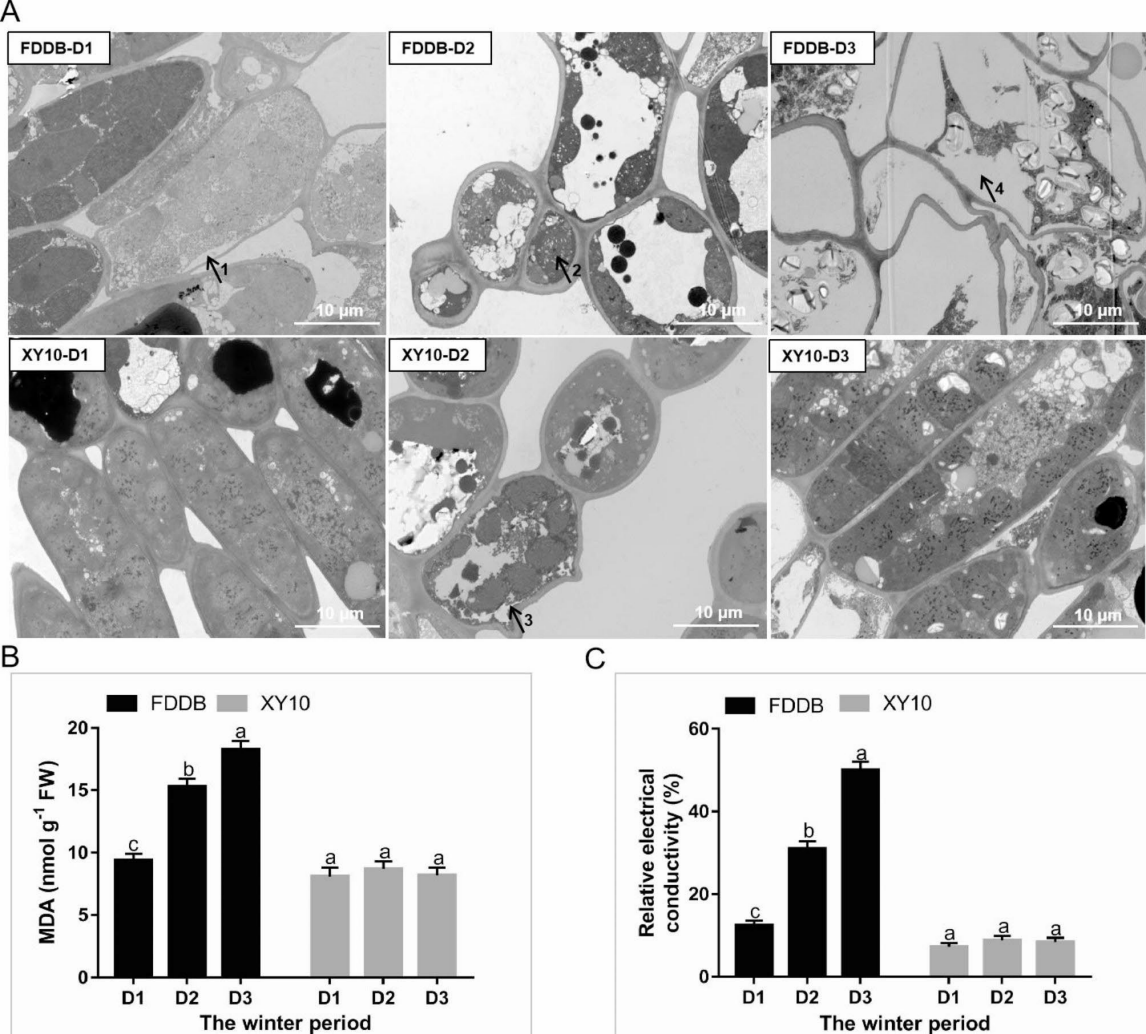
Changes in cell ultrastructure and physiological indexes in leaves of tea cultivars XY10 and FDDB at the overwintering stages of D1, D2 and D3. (**A**) TEM photographs of mesophyll cells. All images were acquired at the same magnification, bars = 10 μm, with Arrow 1 and 3 for the occurrence of plasmolysis, arrow 2 for incomplete chloroplast structure, and arrow 4 for intracellular cavitation. (**B**) MDA content. (**C**) Relative electrical conductivity (REC) level. Data are means ± SD of three biological replicates. Different small letters above the bars indicate significant difference (p < 0.05) among the different overwintering stages for the two tea cultivars

The freezing tolerance of the tea cultivars XY10 and FDDB during natural overwintering was evaluated by analyzing their physiological changes in terms of subcellular structure, malondialdehyde (MDA) (the marker of lipid peroxidation and oxidative stress) content, and relative electrical conductivity (REC) level. The subcellular structure of mesophyll cells of tea samples was analyzed by transmission electron microscopy (TEM) (Fig. 1A). At the first sampling time (D1, December 8) with the temperature at 4 ℃, the leaf of XY10 showed an intact subcellular structure, in contrast to slight plasmolysis in the leaf cells of FDDB (arrow 1), resulting in the better physiological status of XY10 than FDDB at the chill temperature. At D2, the second sampling time with the temperature below 0 ℃, slight plasmolysis could be observed in the leaf cells of XY10 (arrow 3), and FDDB exhibited more severe damage in subcellular structures, as shown by an incomplete chloroplast structure and reduced intracellular material (arrow 2), indicating that the natural freezing stress had less effect on XY10 but caused serious cell damage to FDDB. At D3, the third sampling time with the temperature at 6 ℃ for the recovery of the two cultivars from freezing damage, the ultrastructure of FDDB was observed to be severely damaged with apparently intracellular cavitation (arrow 4), in contrast to complete subcellular structure for XY10, indicating the better recovery of XY10 and more serious injury in FDDB after the rise of temperature. Harmed leaf tissue structure of FDDB at the recover stage was also observed by light microscopy (Supplemental Figure S1). At D3, despite the return of temperature to a higher level, the temperature difference was large between day and night, thus still posing a threat to the growth of tea plants, causing FDDB (which suffered serious damage at D2) to display an even worse physiological status at D3. In XY10, the size of mesophyll cells was observed to decrease at D2 versus D1 and then began to recover at D3 stage, which may be important for the cultivar adaptation to a freezing condition. As important indicators for cell injury, the results of MDA content and REC value were shown to be consistent with the ultrastructure observation results (Fig. 1B and C), both of which were higher in FDDB than in XY10 regardless of the overwintering stages. During the three overwintering stages, both the MDA content and REC level showed an obvious uptrend in FDDB but no significant change in XY10. Together, these results demonstrated that the tea cultivar XY10 was more resistant while FDDB was more sensitive to natural freezing stress.

### Transcriptome profiling analysis of the two tea plant cultivars during natural wintering

The transcriptome profiles of tea leaf samples were investigated by RNA-Seq analysis to understand the gene expression diversity of XY10 and FDDB under three natural wintering stages. A total of 781,576,484 raw data reads were generated, and after filtration and examination of the sequencing error rate and GC content distribution, the clean reads were obtained, ranging from 38.1 to 45.0 million in different libraries (Supplemental Table S1). HISAT2 software was used to align the RNA-Seq reads against the reference genome of tea plant assembled by Wei et al. [6]. A total of 50,525 transcripts were identified in 18 samples and their expression levels were quantified as fragments per kilobase of transcript per million reads mapped (FPKMs). The distribution of the gene expression values (FPKM) of all genes in each sample was displayed in a box plot (Supplemental Figure S2). Additionally, a principal component analysis (PCA) was performed for the gene expressions at different stages in both cultivars (Supplemental Figure S3), where the inter-group samples were dispersed while the intra-group samples were clustered together, suggesting good biological repeatability.

### Identification of differentially expressed genes (DEGs) in different overwintering stages and cultivars

DESeq2 was used for differential expression analysis of the two cultivars during the three overwintering stages, following the steps of standardizing the original readcount, calculating the *p* value, and correcting the multiple hypothesis testing to obtain the padj value [7]. DEGs in each pairwise comparison were screened with |log_2_ FC (fold change) | > 1 and P < 0.05. In order to better analyze the varietal and temporal difference in gene expression patterns, the pairwise comparisons were carried out between the two tea plant cultivars at the same sampling time point and the continuous sampling time points of the same cultivar, generating a total of 7 comparison combinations (Table 1). Compared with FDDB, XY10 had 6490, 5522 and 5675 DEGs at D1, D2 and D3 stages, respectively, indicating their great genetic difference. When comparing D2 with D1, 2252 and 1005 DEGs were identified in FDDB and XY10, respectively, and when comparing D3 with D2, the number of DEGs was 5096 and 3507 in FDDB and XY10, which were 2.3- and 3.5-fold the number of DEGs in the previous comparison combination, respectively. This indicated that the transcriptional responses of the two cultivars were more complex during the temperature recovery stage than in the freezing stage. Note that the number of DEGs in consecutive temporal comparisons was smaller in XY10 than in FDDB, indicating blunter transcriptional changes for the cultivar with stronger freezing resistance.

**Table 1:** The number of DEGs in different pairwise comparisons

	Comparison pair	Number of DEGs	up	down
(1)Comparisons between cultivars at a same sampling time point	D1XY10 vs. D1FDDB	6490	2929	3561
	D2XY10 vs. D2FDDB	5522	2397	3125
	D3XY10 vs. D3FDDB	5675	2365	3310
(2)Comparisons between consecutive sampling time points of the same cultivar	D2XY10 vs. D1XY10	1005	598	407
	D2FDDB vs. D1FDDB	2252	1291	961
	D3XY10 vs. D2XY10	3507	1888	1619
	D3FDDB vs. D2FDDB	5096	2948	2148

### Venn diagrams and GO analysis on specific DEGs associated with varietal diversity

**Fig. 2:**
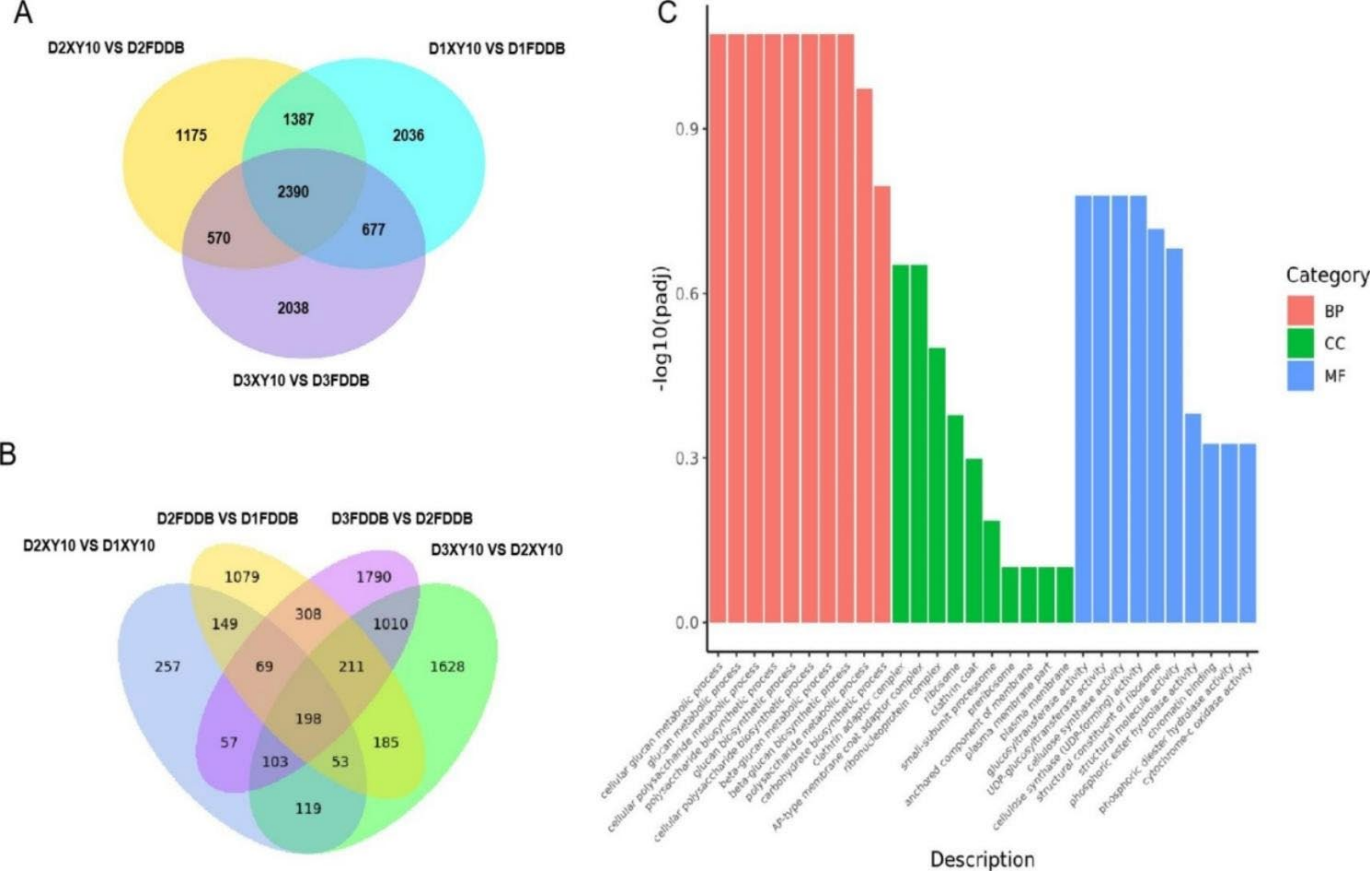
Venn diagrams of overlapped and uniquely expressed DEGs for the pairwise comparisons between the cultivars at the same sampling time point (**A**) and between the consecutive sampling time points of the same cultivar (**B**). (**C**) Top 10 GO terms assigned to different categories by GO analysis of the 1175 DEGs uniquely expressed between the cultivars at D2 but not at D1 or D3 stages

The distribution of DEGs among pairwise comparisons was further investigated by Venn diagrams. Figure 2 A shows the overlap of DEGs in pairwise comparisons between the two cultivars at the same sampling time point. Only the genes with differential expression between the cultivars at the freezing period (D2) could better reflect their genetic differences related to freezing resistance. It was shown that 1175 DEGs in the comparison of D2XY10 versus D2FDDB were not shared by either the comparison of D1XY10 versus D1FDDB or the comparison of D3XY10 versus D3FDDB at both stages. The 1175 specific DEGs were further investigated by GO analysis, which annotated them to 695 GO terms and assigned to three categories: biological process (BP), molecular function (MF) and cellular component (CC) (Fig. 2C). The top 10 GO terms with the smallest padj values were all in the BP category, related to the biosynthesis of cell wall polysaccharides. The next top 6 GO terms were all assigned to the MF category, related with the modification and synthesis of cell wall polysaccharides, including glucosyltransferase activity, UDP-glucosyltransferase activity, cellulose synthase activity, etc. In the CC category, the expression of these DEGs mainly occurred at clathrin adaptor complex, AP-type membrane coat adaptor complex, ribonucleoprotein complex, ribosome, clathrin coat, small-subunit processome, preribosome, anchored component of membrane, and plasma membrane. All these results indicated that the difference between the two cultivars in freezing tolerance might be associated with their difference in cell wall metabolism mechanism.

### Venn diagrams and gene ontology (GO) analysis on specific DEGs associated with temporal variance

The Venn diagram analysis was also conducted to observe the overlap of DEGs for pairwise comparisons between the consecutive sampling time points of the same cultivar. In Figure. 2B, 198 DEGs were seen to overlap among the four pairwise comparisons. These overlapped DEGs were closely related to the response of tea plant to low temperature stress. The 198 DEGs were annotated to 80 GO terms (Supplemental Table S2), including 41 BP, 11 CC, and 28 MF. Specifically, 15 DEGs were annotated into the GO terms of 11 CC categories, with 5 of them described as occurring in cell wall, and the next 4 occurring in the cytoplasmic skeleton. Additionally, 49 DEGs were annotated into the GO terms of 41 BP categories, with 17 of them related to all the metabolic processes responsible for the synthesis and assembly of cell wall polysaccharides, and the remaining entries were scattered in stress response, cell secretion, cell homeostasis, protein modification, signal transduction, biological quality regulation, assembly and synthesis of cellular components, etc. Moreover, 88 DEGs were annotated into 28 terms of MF categories, including cell wall polysaccharide glycosyltransferase activity, oxidoreductase activity, protein modification, substance transport, etc. All these results showed the involvement of the cell wall in the location of gene occurrence, biological processes or gene function, inferring that the cell wall may play an important role in tea plant response to extreme low temperature changes under natural conditions.

### Key DEGs related to transcription factor regulation

The transcription factors (TFs), known as a group of DNA-binding proteins, play a crucial role in plant stress response by regulating gene expression. In the current study, two TFs were filtered by Venn diagram analysis and they may play a fundamental role in tea plant resistance to freezing stress. One was *ERF1B* in the *AP2/ERF* family and the other was *MYC2* in the *bHLH* family (Table 2). The expression of *ERF1B* in both cultivars was higher at D2 than at D1 or D3, with its up-regulation at D2 more obvious in XY10, 3.64-fold higher than the expression of FDDB. The expression of *MYC2* was higher in FDDB and lower in XY10 at D2 relative to D1 and D3, 1.42-fold lower in XY10 than in FDDB at D2 stage. These results suggested that the *ERF1B* and *MYC2* may have a positive and negative regulatory function in tea plant resistance to natural freezing, respectively, and the tea cultivar with strong freezing resistance can maintain high *ERF1B* and low *MYC2* expression under a freezing condition.

**Table 2:** Variations of FPKM values for key DEGs involved in transcription factor regulation and signal transduction at different overwintering stages in different cultivars

Gene ID	Gene name	FDDB(FPKM)			XY10(FPKM)			LOG2FC (XY10 vs. FDDB)		
		D1	D2	D3	D1	D2	D3	D1	D2	D3
CSS0034513	*ERF1B*	0.36	0.83	0.81	0.83	**3.01**	0.68	**1.20**	**1.87**	-0.26
CSS0047466	*MYC2*	1.03	1.33	0.76	0.91	0.49	0.80	-0.18	**-1.42**	0.08
CSS0019921	*CML3*	0.45	**19.53**	0.96	0.14	**4.78**	0.24	**-1.68**	**-2.03**	**-2.01**
CSS0018159	*CML18*	3.31	**20.77**	6.95	3.79	**26.08**	7.83	0.20	0.33	0.17
CSS0033756	*CML23*	4.08	**32.75**	5.84	5.46	**20.73**	3.23	0.42	-0.66	-0.85
CSS0023779	*CML44*	18.86	**25.77**	18.63	13.10	12.14	19.81	-0.53	**-1.09**	0.09
CSS0013301	*CML45*	0.64	**68.95**	0.27	0.64	**27.26**	0.29	0.01	**-1.34**	0.11
CSS0012021	*CDPK3*	1.76	**4.07**	1.34	1.31	1.06	1.93	-0.43	**-1.94**	0.53
CSS0008031	*CDPK28*	1.40	**3.41**	1.12	0.91	0.96	1.14	-0.62	**-1.83**	0.02
CSS0006629	*JAR1*	0.62	**1.99**	0.71	0.58	0.86	0.63	-0.08	**-1.20**	-0.19
CSS0028750	*SAUR36*	2.93	**11.32**	1.78	2.25	**7.57**	1.43	-0.38	-0.58	-0.32
CSS0040775	*NPK1*	30.30	**89.35**	35.95	34.90	**81.45**	30.33	0.20	-0.13	-0.25
CSS0030644	*EDR1*	8.35	**1.24**	7.92	1.87	**0.67**	3.27	**-2.16**	-0.89	**-1.27**
CSS0002306	*MPK3*	3.52	**2.24**	4.60	8.52	**5.76**	9.00	**1.28**	**1.36**	0.97

Note: Bold numbers indicate significantly higher (or lower) FPKM values at D2 stage relative to D1 and D3 in a certain cultivar, or remarkably up-regulated (or down-regulated) gene expression in XY10 versus FDDB at a certain stage

### Key DEGs involved in signal transduction

Signal transduction is a crucial event in plant resistance to low temperature stress. Here, key DEGs were identified to be involved in the signaling systems, such as Ca^2+^ signaling, hormones, and mitogen-activated protein kinases (MAPKs). Ca^2+^ sensor genes, including five *CML* genes (i.e., *CML3*, *CML18*, *CML23*, *CML44*, and *CML45*) and two *CDPK* genes (i.e., *CDPK3* and *CDPK28*), were dramatically up-regulated at D2 stage in both cultivars, but with higher expression in the freezing-sensitive cultivar FDDB than in the tolerant cultivar XY10 (Table 2). The two genes (jasmonic acid-amido synthetase 1 (*JAR1*) and auxin-responsive protein 36 (*SAUR36*)) involved in the hormone signal transduction were identified to have exactly the same change pattern as the Ca^2+^ sensor genes (Table 2), suggesting their similar signal regulatory role to calcium signature. Ca^2+^ and MAPK cascades are important signal transmitting events in plants. The MAPK chain consists of three protein kinases, MAP3K-MAP2K-MAPK, transmitting the upstream signals to downstream kinases to activate various effector proteins through sequential phosphorylation [8]. The three identified MAPK genes belong to the classes of MAP3K (*NPK1*), MAP2K (*EDR1*) and MAPK (*MPK3*), respectively (Table 2). *NPK1* was remarkably up-regulated in the two cultivars at D2 stage, with a FPKM value of 89.35 in FDDB and 81.45 in XY10, respectively, exhibiting a similar expression pattern to the Ca^2+^ sensors, indicating that the Ca^2+^ sensors-induced MAPKs cascades might occur in the response of tea plants to freezing stress. However, the abundance of the downstream *EDR1* and subsequent *MPK3* genes dropped to a lower level (below 10), and their expression was generally suppressed at D2 stage in tea cultivars, suggesting they may function as a negative regulator in signal transduction.

### Key DEGs associated with cell wall

**Fig. 3:**
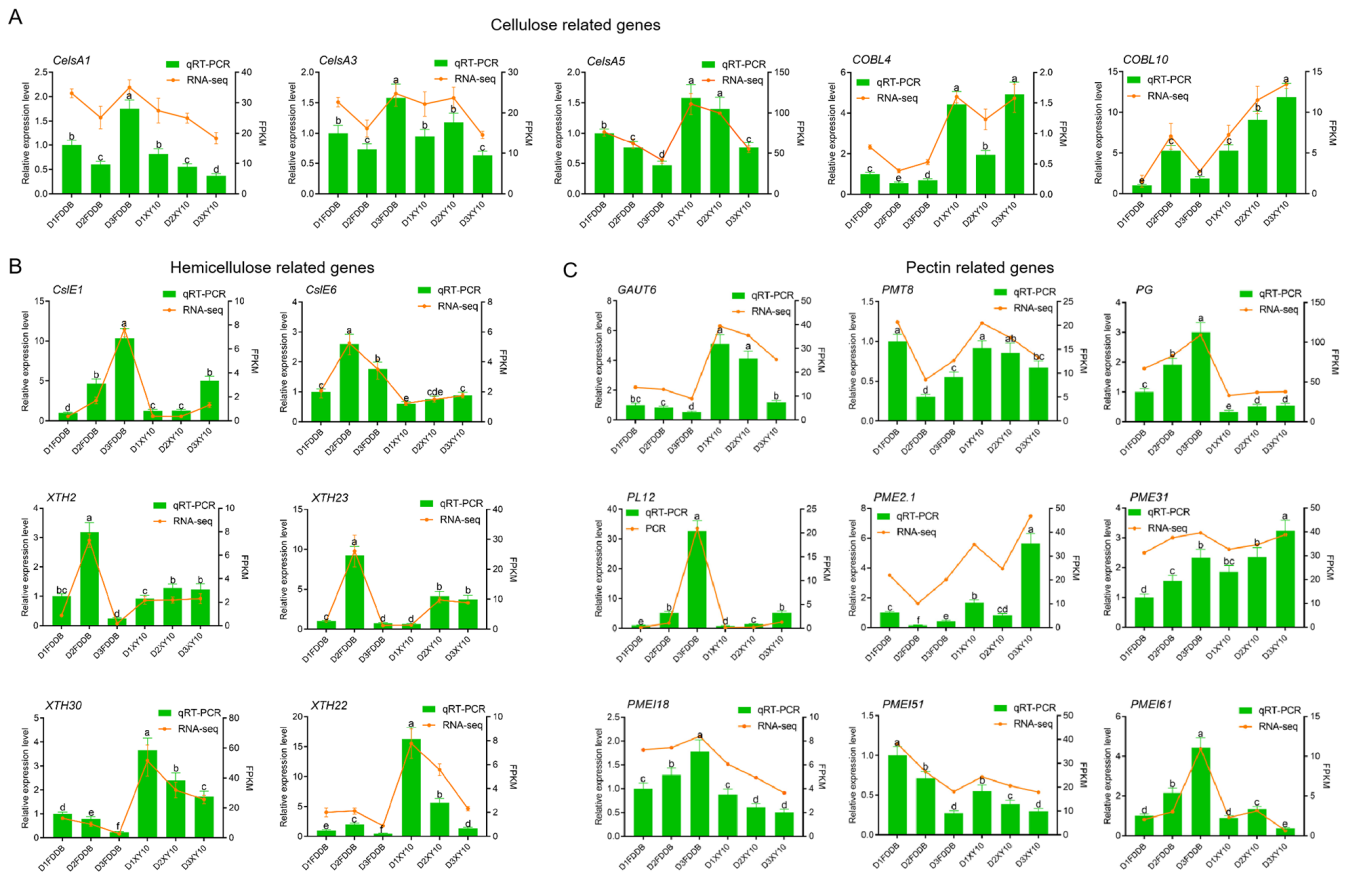
Transcriptional changes of key DEGs involved in the biosynthesis and recombination of cell wall polysaccharides in the two tea cultivars during wintering. The expression level of DEGs related to the cellulose (**A**), hemicellulose (**B**) and pectin (**C**) as validated by qRT-PCR. Data are expressed as mean ± SD of three biological replicates. Different small letters above the bars indicate significant difference (p < 0.05) among the different sample groups

GO term analysis indicated that the cell wall was heavily involved in tea plant resistance to natural freezing stress, so transcriptional analysis was further performed for the specific DEGs involved in the synthesis, degradation and structural modification of cell wall polysaccharides (Supplemental Figure S4). The expression patterns of cell wall genes were also validated by quantitative real-time PCR (qRT-PCR), and the results were highly consistent with the FPKM values from the RNA-Seq data, confirming the reliability of our transcriptome data (Fig. 3A-C).

Cellulose, hemicellulose and pectin are the three main polysaccharides forming the basic structure of plant cell wall. We identified three cellulose synthase genes (*CesA1*, *CesA3* and *CesA5*) encoding cellulose biosynthesis and two *COBLs* (*COBL4* and *COBL10*) involved in the modulation of cellulose deposition and orient cell expansion in plant [9]. At D1 stage, the expression of *CesA5* was significantly higher in XY10 compared with FDDB, although the expression of *CesA1* and *CesA3* did not differ across varieties, which implied that XY10 may own stronger cellulose synthesis capacity than FDDB at the chilling condition. In comparison to D1 stage, all the cellulose synthases were remarkably down-regulated in FDDB while fluctuated slightly in XY10 at D2. The expression of two *COBLs* were higher in XY10 than in FDDB regardless of time points. Additionally, their expression patterns were inconsistent over time, with the expression being inhibited for *COBL4* and promoted for *COBL10* under natural freezing stress in both cultivars. These results indicated that the cellulose amount was more steadily regulated and the orientation of cellulose microfibrils was more flexibly modulated in the antifreeze tea cultivar under a freezing condition.

Cellulose synthase-like (*Csl*) genes belonging to the same gene superfamily of *CesA* are involved in the formation of hemicellulose [10]. Two *Csl* genes (*CslE1* and *CslE6*) were identified, and their expression was overall higher in FDDB than in XY10. With the extension of overwintering time, their expression was elevated in both cultivars, with a more significant change in FDDB. Xyloglucan is the main type of hemicellulose in dicotyledonous plants, such as tea. Four xyloglucan endotransglucosylases/hydrolase (*XTH*) genes (*XTH2*, *XTH23*, *XTH30* and *XTH22*) were identified through large-scale data analysis. Among them, the expression of *XTH2* and *XTH23* was enhanced at D2 in both cultivars, with a more significant change in FDDB. However, the expression of *XTH30* and *XTH22* was suppressed, with a more obvious change in XY10. These results revealed that the expression of hemicellulose related genes varied with time and cultivar.

Pectin is a class of acid heteropolysaccharides with galacturonic acid as the main component in cell wall. Four genes associated with pectin abundance were identified, including *GAUT6*, *PMT8*, *PG* and *PL12*. Among them, the galacturonosyltransferases (*GAUTs*) and pectin methyltransferases (*PMTs*) are key genes involved in pectin synthesis, while the polygalacturonases (*PGs*) and pectin lyases (*PLs*) are crucial to pectin degradation [11]. Compared with FDDB, XY10 showed higher expression in *GAUT6* and *PMT8*, while lower expression in *PG* and *PL12*, demonstrating stronger pectin biosynthesis and weaker pectin degradation in XY10 than in FDDB. The expression was suppressed for *GAUT6* and *PMT8* but enhanced for *PG* and *PL12* under natural freezing stress at D2 in both tea cultivars, and the changes were more evident in FDDB than XY10, implying that freezing stress could cause a decrease in pectin biosynthesis and an increase in its degradation, and the effect was weaker in freezing resistant tea cultivar. Pectin methylesterification, an important factor affecting cell wall structure, was regulated by pectin methylesterases (PMEs) which can catalyze the demethylation of pectin and pectin methylesterase inhibitors (PMEIs, with the function of restraining the activity of PMEs). In the present study, two *PME* genes (*PME2.1* and *PME31*) and three *PMEI* genes (*PMEI18*, *PMEI51* and *PMEI61*) were identified. Compared with FDDB, XY10 had a higher expression level of *PMEs* and a lower expression level of *PMEIs*, indicating a higher demethylesterification of pectin in XY10 than in FDDB. However, with winter progressing, either *PMEs* or *PMEIs* showed an inconsistent expression pattern, indicating that the actual regulation process of *PMEs* and *PMEI* on pectin methylesterification in tea cultivars during overwintering was complicated.

### Determination of cell wall polysaccharide contents

**Fig. 4:**
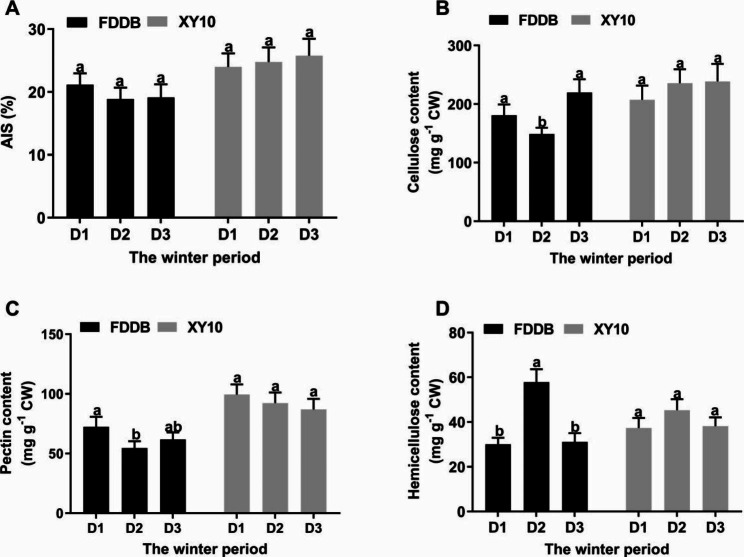
Content changes of cell wall (**A**) and its polysaccharide components, including cellulose (**B**), pectin (**C**) and hemicellulose (**D**) in leaves of tea cultivars XY10 and FDDB at the overwintering stages of D1, D2 and D3. The cell wall was quantified as alcohol-insoluble solid (AIS). Polysaccharide content was calculated based on the cell wall (CW). Data are expressed as mean ± SD of three biological replicates. Different small letters above the bars indicate significant difference (p < 0.05) among the different overwintering stages for the two tea cultivars

The alcohol-insoluble solid (AIS) was isolated from different tea leaf samples for quantification of cell wall content and its polysaccharide components (Fig. 4). Generally, the contents of cell wall and its polysaccharides were higher in XY10 than in FDDB, indicating that XY10 had a tighter or thicker cell wall structure. With the extension of wintering time, it was observed that the content changes of cell wall polysaccharides were not significant in XY10, while FDDB had a remarkable decrease in the contents of cellulose and pectin and an obvious increase in hemicellulose content at D2 stage, demonstrating that the cell wall structure was more stable in the freeze-resistant tea cultivar during overwintering.

## Discussion

The physiological results showed that the freezing-tolerant tea cultivar XY10 outperformed the freezing-sensitive cultivar FDDB in the freezing stage and the recovery stage (Fig. 1). When plants encounter freezing stress, ice crystals will form first and gradually grow and diffuse from the extracellular to the intracellular spaces, leading to protoplast dehydration, ice squeeze-induced cellular mechanical damage and osmotic stress induced by membrane lipid peroxidation due to ROS attack caused by the former two stresses [12]. This indicated that the ability to maintain a stable and unharmed cell structure in cell wall [13] and cell membrane [14] is essential for plant survival under freezing conditions, which can prevent the propagation of outer ice crystals into the cell. TEM observation indicated that XY10 had a more complete subcellular structure than FDDB during the freezing period (Fig. 1). The freezing-tolerant tea cultivar could maintain a good physiological state under freezing stress, which is probably due to a greater structural resistance of its cells, maintaining the osmotic balance and the functioning of the tissues, allowing it to achieve a better recovery in the later period. Interestingly, the cell size was observed to decrease for the freezing-tolerant tea cultivar at D2 stage, which may be related to the adaptation of XY10 to sub-zero low temperature by changing the cell wall structure. As previously reported, making the cell wall less porous and compact by recombination of its polysaccharides is an important strategy for plant to prevent the propagation of outer ice crystals [15], which may restrict cell elongation and diminish its size.

The statistics on the number of DEGs identified by transcriptome profiling analysis revealed that the freezing-resistant tea cultivar exhibited blunter transcriptional changes than the freezing-sensitive cultivar (Table 1). This was consistent with the gene expression characteristics of tea plant response to cold stress reported by Wang et al. [4], who found retardation of gene expression in tea cultivars with stronger cold resistance. Additionally, Li et al. [5] found that the peak time for the expression of most DEGs was earlier in the cultivar with greater cold resistance. This well explained our present finding that the peak time of gene expression in response to temperature changes in the freezing-resistant tea cultivar may be earlier, causing fewer DEGs observed relative to the sensitive cultivar. Based on the Venn diagrams, we selected 1175 and 198 key DEGs closely associated with varietal specificity and temporal variance, respectively (Fig. 2). Interestingly, GO term analysis of these two groups of key genes indicated that the cell wall may play a vital role in tea plant resistance to natural freezing stress and is a key factor for the freezing tolerance discrepancy between the two tea cultivars (Fig. 2 and Supplemental Table S2). Plant cell wall is mainly composed of polysaccharides as cellulose, hemicellulose, and pectin, with a complex structure wrapped outside the plant cells. As the first barrier for plant cells to contact the environment, cell wall plays a very important role in the process of plant resistance to various stresses [16].

Celluloses are present as microfibrils which consist of dozens of hydrogen-bonded β-1,4 glucan chains and are cross-linked with each other to form the cell wall skeleton structure [17]. The deposition amount and orientation of cellulose microfibrils are the two major aspects affecting the construction of cellulose array, which can produce an important impact on cell wall morphogenesis and defense [9]. In this study, we identified three *CesA* genes and two *COBL* genes related to cellulose biosynthesis and deposition, respectively (Fig. 3A). Their higher expression in XY10 than FDDB at the freezing stage suggested that the freeze-resistant tea cultivar may accumulate more celluloses in response to natural freezing, which was verified by the results of cellulose content determination (Fig. 4). The mutants with knocked-out *CesAs* or *COBLs* in some plants such as bread wheat [18], flax [19] and rice [20] were reported to exhibit obvious reduction in cellulose content, coupled with impaired cell wall structure and mechanical strength, as well as limited plant growth. The variations in the freezing tolerance of different tea cultivars can be attributed to the influence of cellulose accumulation and cell wall integrity regulated by *CesAs* and *COBLs*.

Pectin, as another important type of cell wall polysaccharide, performs the most complex and flexible functions in plant defense by influencing the cell wall integrity, rigidity, porosity and so on [21]. Maintaining a stable and even an enhanced pectin level seems to be a key element for the plant to cope with cold stress. For example, in the leaves of winter oil-seed rape, the pectin content was observed to increase during plant acclimation in the cold, and it was especially higher in the cold-tolerant relative to the cold-sensitive line [22]. In maize leaves, the pectin level was not affected in the chilling-tolerant line, but was significantly reduced in the chilling-sensitive line under low temperature stress, which was associated with more severe cellular damage in the chilling-sensitive line [23]. In tea plant response to other abiotic stresses (i.e. ionic stresses), the pectin biosynthesis was shown to be promoted under the regulation of pectin genes like *GAUT* [24, 25]. However, there has been no report available on the change of pectin level in tea plant under low temperature stress. In the current study, we found that XY10 had higher expression in the genes related to pectin biosynthesis and lower in the expression of genes involved in pectin degradation in response to freezing stress (Fig. 3C), resulting in higher accumulation of pectin materials as confirmed by pectin content detection results (Fig. 4). This indicated that the freeze-resistant tea cultivar can maintain a sufficient abundance of pectin to ensure the integrity, thickness, and normal function of the cell wall in a freezing condition.

The expression of genes involved in pectin methyl esterification modification revealed that the pectin demethylesterification process occurred more frequently in XY10 than FDDB (Fig. 3C). De-methylesterified pectin was found capable of cross-linking Ca^2+^ and forming a structure with reduced cell wall porosity, thus enhancing the freezing survival of Allium fistulosum [26]. Therefore, the stronger demethylesterification of pectin in the freeze-resistant tea cultivar may contribute to the formation of a compact cell wall structure.

The speed and degree of ice crystal propagation in tea plant cells is an important factor for the degree of freezing damage [27]. The cell wall is an important protective barrier to prevent ice crystals from entering the cell interior, and its stable or even strengthened structure under freezing conditions is of great significance to the success of resistance to freezing damage [15]. Based on the above analysis, the freeze-resistant tea cultivar can be assumed to maintain higher cell wall integrity and tightness, thus effectively preventing ice crystal invasion and enabling its survival under a freezing condition.

The hemicellulose is defined as a polysaccharide with a neutral sugar skeleton and a branching structure. In this study, several hemicellulose-related key genes were identified, including two *Csls* and four *XTHs*, whose expression was irregular and inconsistent over time and across breeds (Fig. 3B), indicating the complexity of actual regulation. Xyloglucan is the main type of hemicellulose in dicotyledons such as tea plant, which plays an important role in regulating the elasticity and mechanical strength of cell wall by crosslinking cellulose [28], and the cellulose-xyloglucan network is regulated by *XTHs* [29]. Proteins encoded by genes of *XTH* family may have the activities of hydrolyzing xyloglucan β-1,4 glycosidic bonds or transferring xyloglucan fragments between xyloglucan molecules [30], and the four *XTHs* (*XTH2*, *XTH23*, *XTH30* and *XTH22*) identified here belong to the latter, indicating the cell wall recombination may be regulated by *XTHs* in tea plant response to freezing stress. As important regulatory genes for cell wall recombination, the *XTHs* have been shown to be widely involved in plant resistance to external stresses, including low temperature stress [31, 32]. However, their involvement in tea plant response to the low temperature stress has not yet been reported, and our study provided candidate genes for further related research. Despite irregular gene expression, the hemicellulose content was remarkably increased and higher in FDDB than in XY10 at D2 stage (Fig. 4). Hemicellulose has been reported to promote cell wall expansion by preventing cellulose aggregation [33]. This suggested that the cell wall might be induced to expand in FDDB at D2 stage. Considering its inhibited cellulose amount, the expansion of FDDB cell wall was probably excessive and distorted due to insufficient cellulose supply during the freezing damage period.

Mehrotra et al. [34] has proposed that when the freezing signal is perceived and transduced by signal transduction proteins, transcription factors (TFs) will be activated to regulate the expression of function genes, resulting in physiological changes. Among the key genes identified, some are shown to be involved in signal transduction and transcriptional regulation. Calcium signaling is the most important and initial signaling system in plant response to low temperature stress [35]. Low temperature stimulus would trigger Ca^2+^ signature, which is characterized by changes in Ca^2+^ concentration in the cytoplasm and transmitted by various Ca^2+^ sensors like Ca^2+^-dependent protein kinases (CDPKs), calmodulins (CaMs), CaM-like proteins (CMLs), calcineurin B-like proteins (CBLs), and CBL-interacting protein kinases (CIPKs) [35]. The involvement of these Ca^2+^ sensor genes in low temperature stress response has been well documented in plants including tea plant, but most studies mainly focused on plant response to cold stress, paying little attention to how these genes function in an extreme sub-zero freezing process [5, 36]. In a transcriptomic analysis on the cold adaption process of different tea cultivars, Li et al. [5] found the up-regulation of several Ca^2+^ sensor genes (*CaMs*, *CDPKs*, *CBLs*, *CMLs* and *CIPKs*) specifically in the tea cultivar with higher cold tolerance. In the current study, we identified five *CML* genes (*CML3*, *CML18*, *CML23*, *CML44*, and *CML45*) and two *CDPK* genes (*CDPK3* and *CDPK28*) (Table 2), indicating the involvement of fewer types of Ca^2+^ sensor genes in tea plant response to natural freezing stress, different from the case of cold adaptation process in tea plant. The expression of the Ca^2+^ sensor genes was remarkably increased in both cultivars at D2 stage, suggesting that they may play crucial roles in freezing signal transduction in tea plant. However, their expression was lower in the freeze-resistant cultivar than in the freeze-sensitive cultivar, which was contrary to our expectations. One possible explanation is that the freeze-sensitive cultivar could produce more reactive oxygen species (ROS) due to freezing damage [4], which was reported to facilitate the release of cellular Ca^2+^ and trigger the signature [37], thus stimulating higher gene expression of Ca^2+^ sensors in the freeze-sensitive cultivar. Moreover, we also identified several other signal transduction genes belonging to the signal systems like hormones and MAPKs, most of which showed the same change pattern as the calcium signaling genes. Some Ca^2+^ sensors like *CDPKs* are reported to be capable of connecting Ca^2+^ transients to downstream phosphorylation events by activating the MAPKs cascades [38]. Thus, these signaling genes may play a synergistic role, but their relationship to signaling transduction regulation in tea plant response to freezing stress is worthy of further study.

TFs, the important transcriptional regulators of gene expression, are classified into different families based on the DNA binding domains. Numerous TFs in the families of MYB, NAC, bZIP, AP2/ERF, bHLH, WRKY, etc. have been shown to be involved in plant response to low temperature stress [34, 39]. Among them, a transcriptional cascade widely known as ICE1-CBF-COR is considered as the core controller of cold acclimation. The TF ICE1(inducer of CBF expression 1) and its homolog ICE2 encode a MYC-type basic helix-loop-helix (bHLH) TF, which controls the expression of CBF (C-repeat-binding factor) genes to activate the expression of downstream COR (cold-responsive genes). The CBF genes belong to the TF superfamily AP2/ERF (APETALA2/Ethylene-Responsive Factor), where the TFs can bind to the GCC box (AGCCGCC) in the promoters of ethyleneinducible genes that encode stress-responsive proteins [40]. In tea plant, several CsAP2/ERF genes have been shown to participate in cold response regulation [3, 41]. Recently, Zhang et al. [42] have reported that the expression level of CsCBFs was strongly positively correlated to cold tolerance in 16 tea cultivars, and a CsCBF gene (CsCBF5) was verified as a positive regulator in cold stress response. Here, we identified a CsAP2/ERF gene (*ERF1B*) and a *Cs*bHLH gene (*MYC2*), which are responsive to natural freezing stimulation and associated with cultivar specificity (Table 2), and they may play a central regulatory role in activating the downstream functional genes, such as cell wall-related genes. The expression of *MYC2* marked a complete reversal of the *ERF1B* trend. A possible explanation is that *MYC2* is probably an upstream gene that negatively regulates *ERF1B*, because the *MYC*-type genes have been reported to regulate the *CBF* genes which belong to the same family as *ERF1B* [40].

## Conclusion

**Fig. 5:**
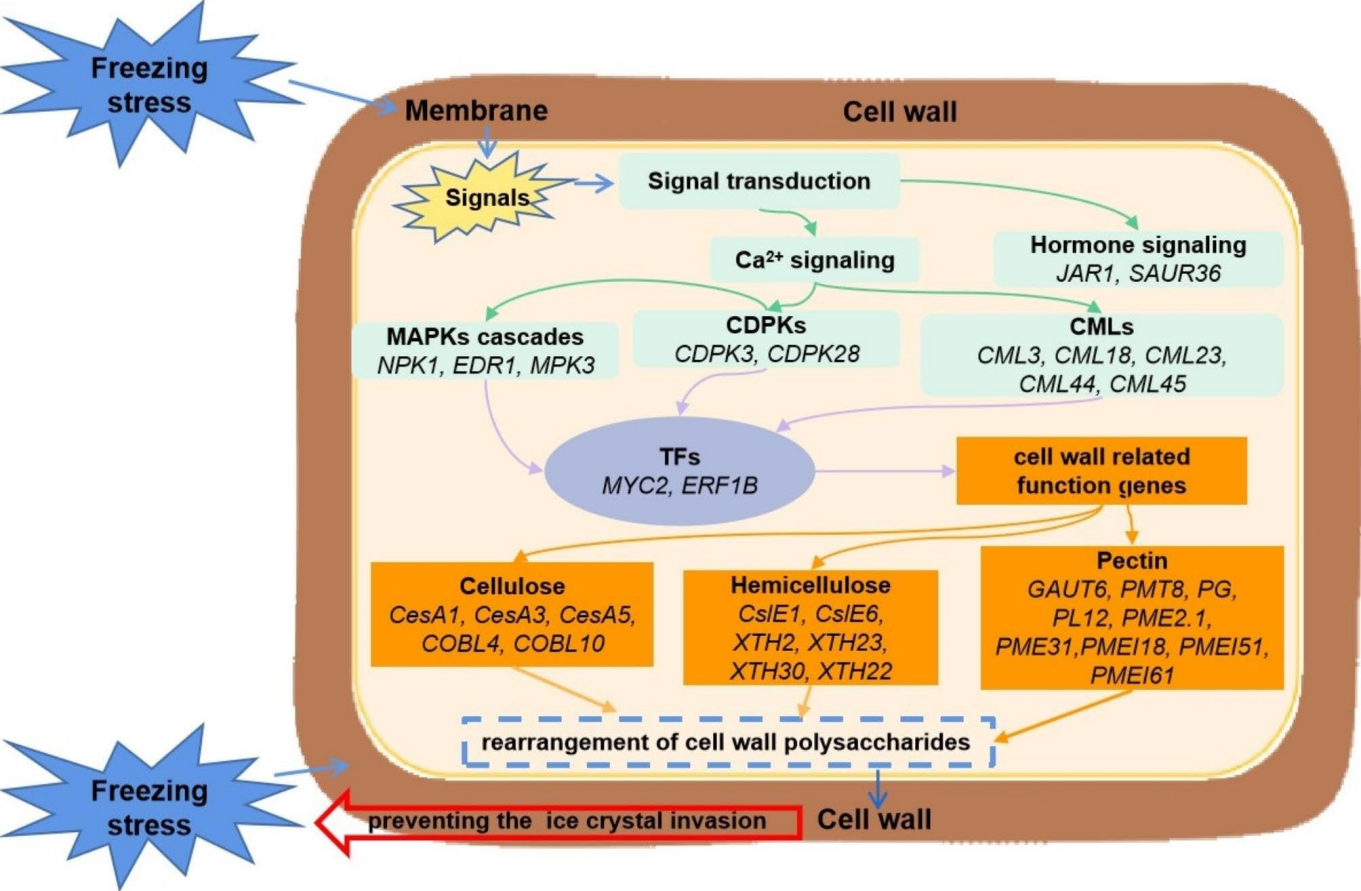
A speculative model for the cooperative regulation of the identified key genes related to signal transduction, transcription factor regulation and cell wall in tea plant response to natural freezing stress

This study investigated the physiological and genetic regulatory changes of a freeze-tolerant (XY10) and a freeze-sensitive (FDDB) tea cultivar during overwintering. The difference between the two tea cultivars in freezing tolerance was shown to be closely related to the cell wall structure variations under the regulation of vital cell wall candidate genes. A model of tea plant responding to natural freezing stress was constructed in Fig. 5. The natural freezing stress could induce the activation of the signal transduction system associated with Ca^2+^ signaling, MAPKs cascades and hormone signaling, up-regulating the expression of the TF *ERF1B*, then regulating the expression of cell wall-related genes and finally altering the cell wall structure. The freeze resistant tea cultivar could maintain a complete and compact cell wall structure, which may effectively block the transfer of ice crystals through the cell and prevent the tea plant from freezing damage. Our results facilitate the understanding of how tea plant adapts to natural freezing temperatures by regulating gene expression and cell wall properties. Future research can further explore the specific molecular functions of the identified cell wall genes and other key genes related to the freezing resistance of tea plants.

## Materials and methods

### Plant materials and sampling

**Fig. 6:**
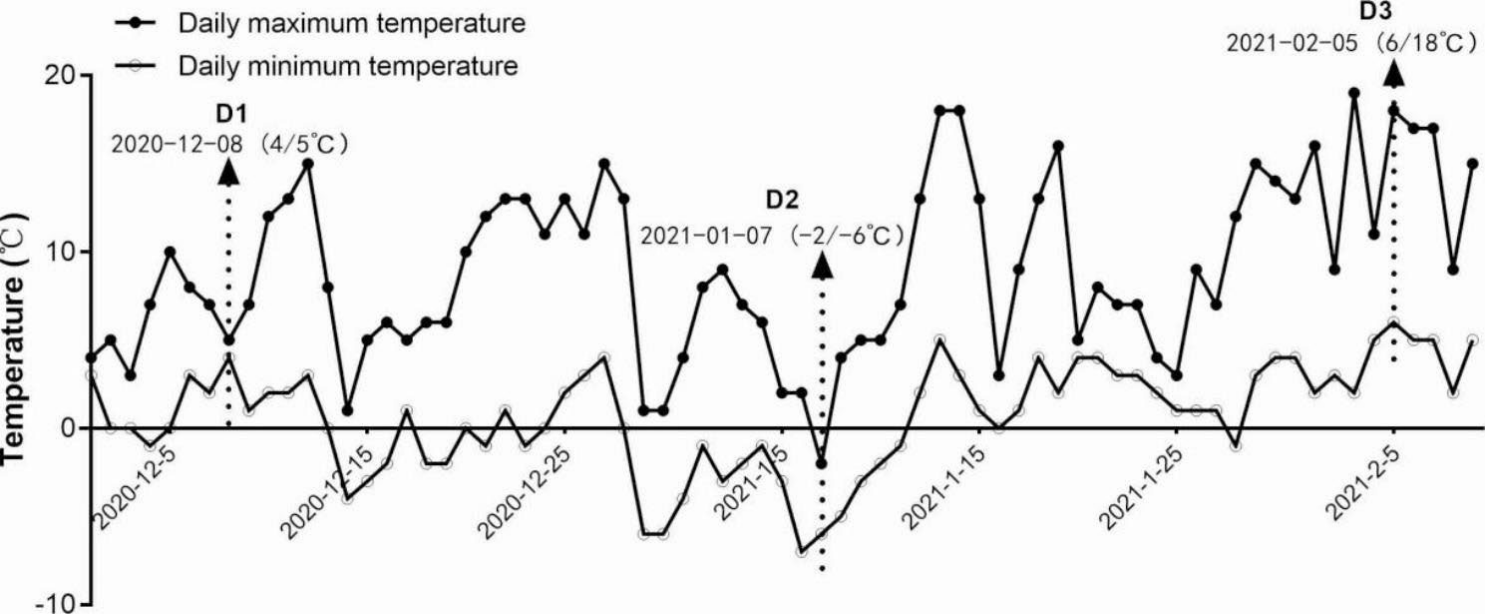
The maximum and minimum air temperatures of Xinyang City from December to February in 2020–2021, where three sampling time points were marked

Ten-years-old tea plants XY10 and FDDB were planted in Pingqiao District, Xinyang City, Henan Province, China (N32°6 ‘, E114°7 ‘, H101 m). The freezing-resistant cultivar XY10 was bred from Xinyang-group species in 1988 by Xinyang Tea Experimental Station (Henan, China). FDDB native to Bailiu Village, Diantou Town, Fuding City (Fujian, China) has been widely cultivated in various tea production regions in China and thus was used as the control cultivar in this experiment. To better observe the changes of tea plants during freezing stress, one bud and two leaves of the two tea cultivars were sampled three times in the freezing occurrence stage and the period before and after the freezing weather in winter. As shown in Fig. 6, the first sample (D1) was taken during the low temperature period above zero, the second sample was collected when the temperature dropped to the temperature below zero, and the third sampling was done after the temperature recovery to warm above 6 °C.

### Light microscopy and TEM observation

For light microscopy, the second leaf of the tea plant approximately 1 cm^2^ in the middle were pre-fixed in formalin-aceto-alcohol (FAA) for 24 h, dehydrated with dehydrator (JJ-12 J, China), washed with xylene, and then paraffin-embedded using a histocentre embedding machine ( JB-P5, China). Next, samples were sectioned with a microtome (RM2016, China) at 5 μm thickness and stained with fast green and safranin for observation under a light microscope (Nikon Eclipse CI, Nikon DS-U3, Japan). The leaf ultrastructural changes in different samples were characterized by TEM. Briefly, small tea leaf blocks from the second leaf of the tea plant no more than 1 mm^3^ were successively fixed with 2.5% glutaraldehyde (pH 7.4) and 1% OsO4 (pH 7.4) at room temperature, followed by dehydration with an ascending gradient of ethanol-water and acetone-ethanol solutions, penetration and embedding by the resin EMBed 812. Next, the resin blocks were polymerized in an oven at 65 ℃, cut to 60–80 nm thin pieces on the ultramicrotome, placed on 150-mesh cuprum grids and stained by 2% uranium acetate and 2.6% lead citrate in sequence. Finally, the cuprum grids were observed and photographed under TEM (HT7800/HT7700, HITACHI, Japan).

### Physiological index detection

The malondialdehyde (MDA) content was detected using the thiobarbituric acid (TBA) method as described by Dhindsa et al. [43], and the relative electrical conductivity (REC) was assayed following the method reported by Wang et al. [4].

### RNA extraction and sequencing

The total RNA was extracted from the leaf samples of the two tea cultivars under three different overwintering stages (D1, D2, D3) using the Quick RNA Isolation Kit (Huayueyang Biotechnology Co., Ltd., Beijing, China), and the RNA integrity was assessed using the RNA Nano 6000 Assay Kit of the Bioanalyzer 2100 system (Agilent Technologies, CA, USA). Then, the mRNA was collected from the total RNA by Oligo (dT) magnetic bead enrichment, randomly interrupted with divalent cations in the NEB fragmentation buffer and sent for library construction. The libraries were tested using the Agilent 2100 bioanalyzer (Agilent Inc., USA) and sequenced on an Illumina Novaseq 6000 platform (Illumina Inc., USA) with paired-end reads generated for each sample. After filtering the original sequencing data and checking the sequencing error rate and GC content distribution, clean reads were obtained for subsequent analysis.

### DEGs identification and GO enrichment analysis

To acquire the genetic information, the clean reads were first matched to the reference genome assembled by Wei et al. [6] using the HISAT2 software (version 2.0.5, parameter -p4 --dta -t --phred33). Subsequently, the number of reads covered by each gene from transcription starting site (TSS) to end of 3’UTR was counted according to the position information of gene alignment on the reference genome. On this basis, the expected number of fragments per kilobase of transcript sequence per million base pairs sequenced (FPKM) was calculated to represent the gene expression value of RNA-seq. The DEGs were then identified by using DESeq2 (version 1.16.1) to calculate the padj values (corrected p-values) for multiple hypotheses tests based on the false discovery rate (FDR) [7]. A |log2 FC (fold change) | > 1 and a padj < 0.05 were used as the standard. The Venn diagrams were further drawn online (https: //magic.novogene.com) to screen for the key DEGs shared or unique to several comparison combinations. Additionally, GO enrichment analysis was performed on the selected key DEGs by using the clusterProfile software (version 3.4.4) with the threshold of padj < 0.05 to discover the key biological processes.

### qRT-PCR verification of DEGs related to cell wall metabolism

To verify the accuracy of RNA-seq data, the relative gene expression of twenty key DEGs involved in cell wall metabolism was detected by qRT-PCR. The experiment was carried out as described in our previous study with the Glyceraldehyde 3-phosphate dehydrogenase (GAPDH) used as an internal control [25], and the primers are listed in Supplemental Table S3.

### Quantification of cell wall polysaccharides

The contents of cell wall polysaccharides were determined according to our previous methods [44]. Briefly, the cell wall materials of tea leaf samples were collected as AIS, freeze-dried and weighed. The AIS was successively extracted with 50 mM Na_2_CO_3_ and 4 M KOH to obtain the pectin and hemicellulose fraction, respectively, and the remaining residue was assigned as cellulose materials. The pectin content was quantified by detecting the content of galacturonic acid in the pectin fraction using the m-hydroxybiphenyl method [45]. The hemicellulose and cellulose contents were quantified by the glucose content in their respective extraction fractions using the anthrone-sulfuric acid method [46].

### Statistical analysis

All data are presented as mean ± standard deviation (SD) of three biological replicates and statistically analyzed by one-way analysis of variance (ANOVA) followed by Duncan’s test using SPSS 19.0 software with a *p* value < 0.05 considered significant.

### Electronic supplementary material

Below is the link to the electronic supplementary material.

Supplementary Material 1

## Data Availability

The datasets generated and analyzed during the current study are available in the [National Center for Biotechnology Information (NCBI) SRA] repository, [accession number: PRJNA976482. persistent web link: https://www.ncbi.nlm.nih.gov/sra/PRJNA976482 ].
