# Supplementary material for: Physiological and transcriptomic analyses reveal tea plant (Camellia sinensis L.) adapts to extreme freezing stress during winter by regulating cell wall structure
Source: BMC Genomics. 2023 Sep 20;24:558. doi: 10.1186/s12864-023-09670-1 (PMC10512626; doi:10.1186/s12864-023-09670-1)
Supplement: Supplementary file 1 — Supplementary Material 1 [file 12864_2023_9670_MOESM1_ESM.docx]

**Supplementary material**

**
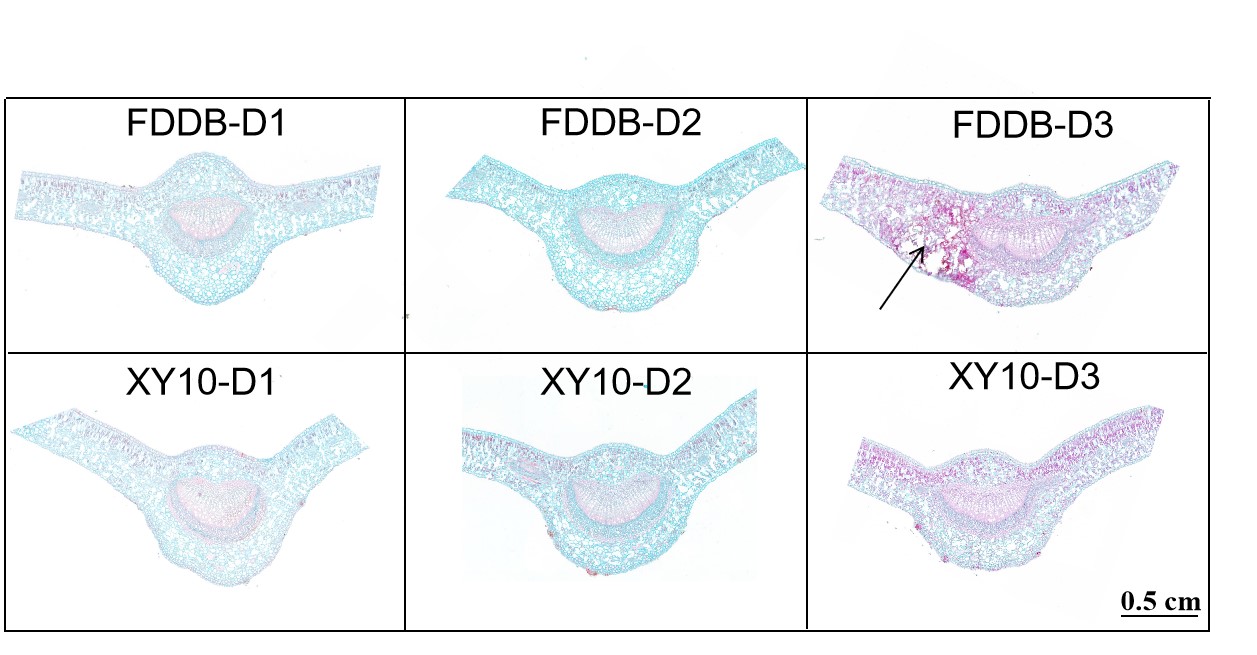
**

**Figure S1** Light microscopy images of tea leaves stained with safranine and solid green in different tea varieties during the overwintering. The black arrow indicates that the leaf tissue of FDDB was severely damaged during the D3 period.


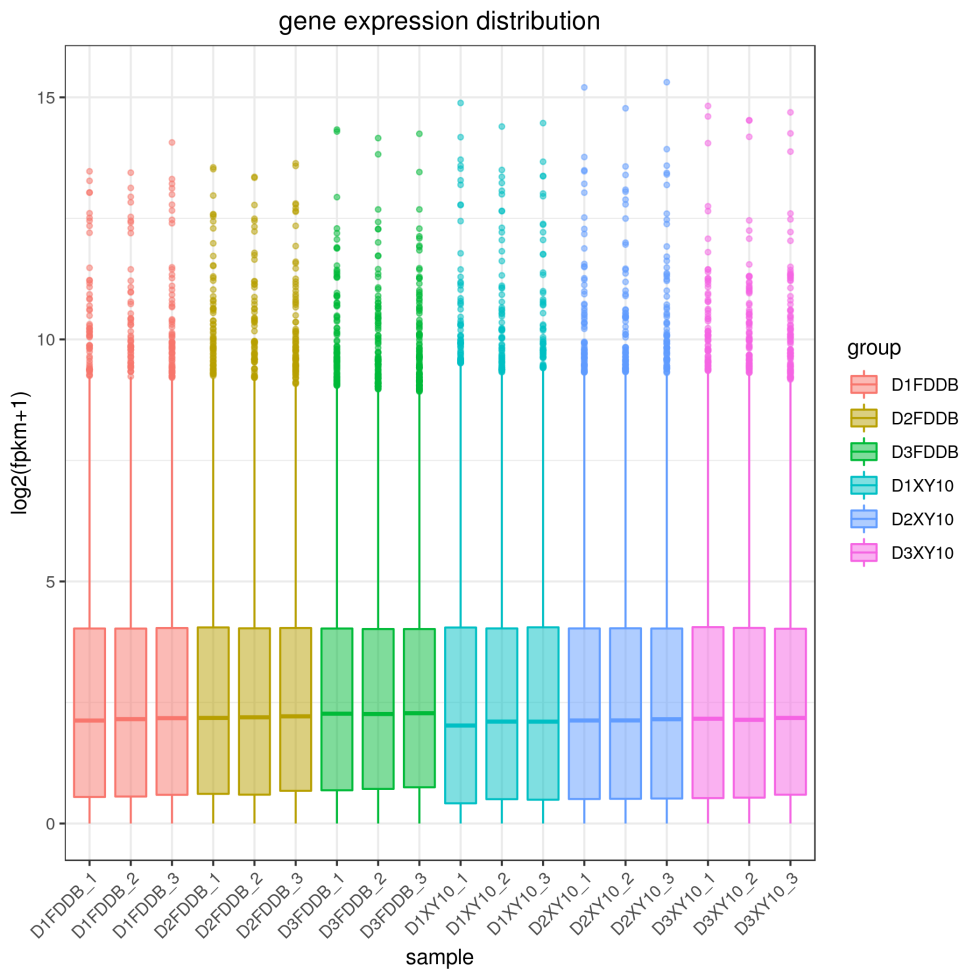


**Figure S2** Box plot of gene expression distribution of different samples.


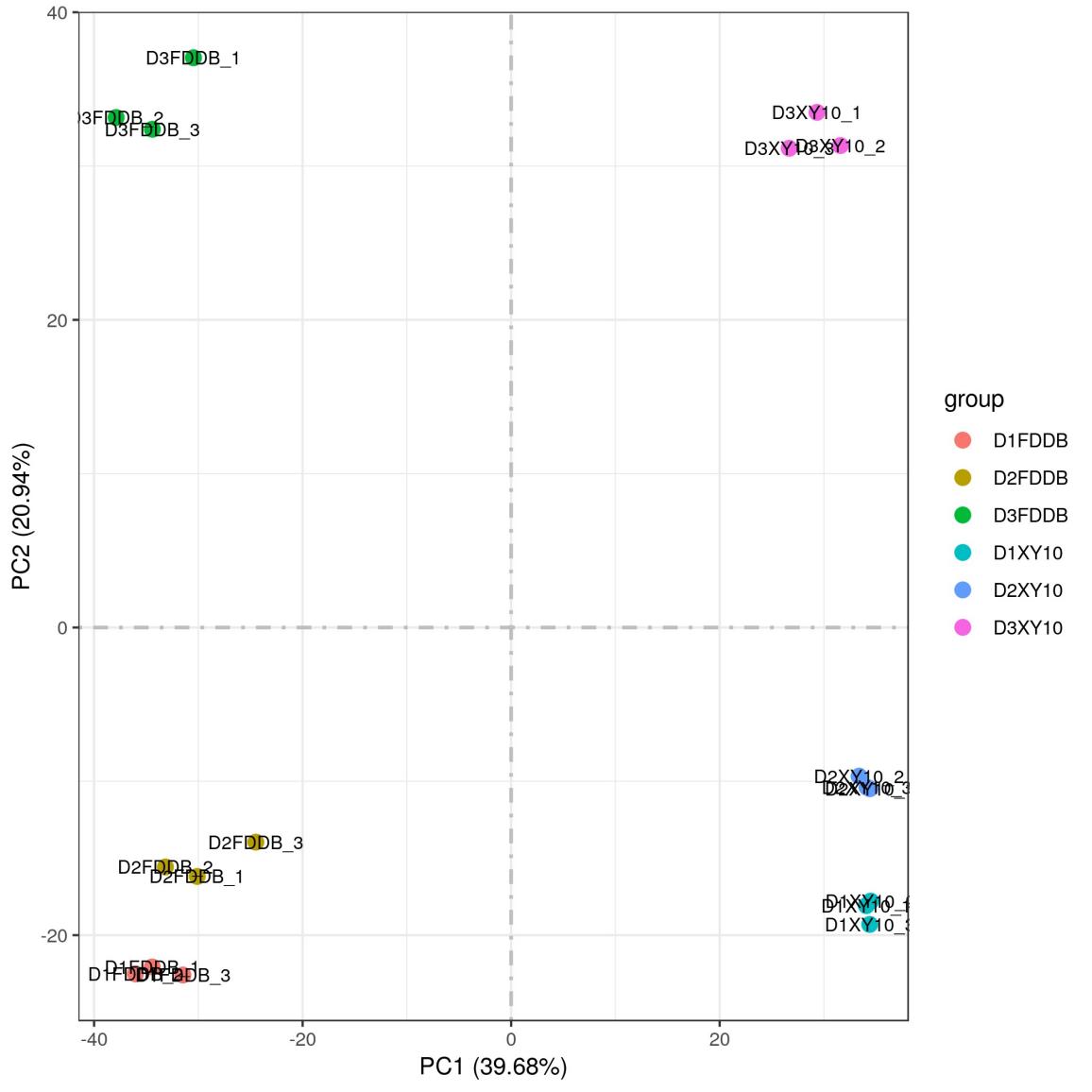


**Figure S3** Principal component analysis of gene expression for different samples.


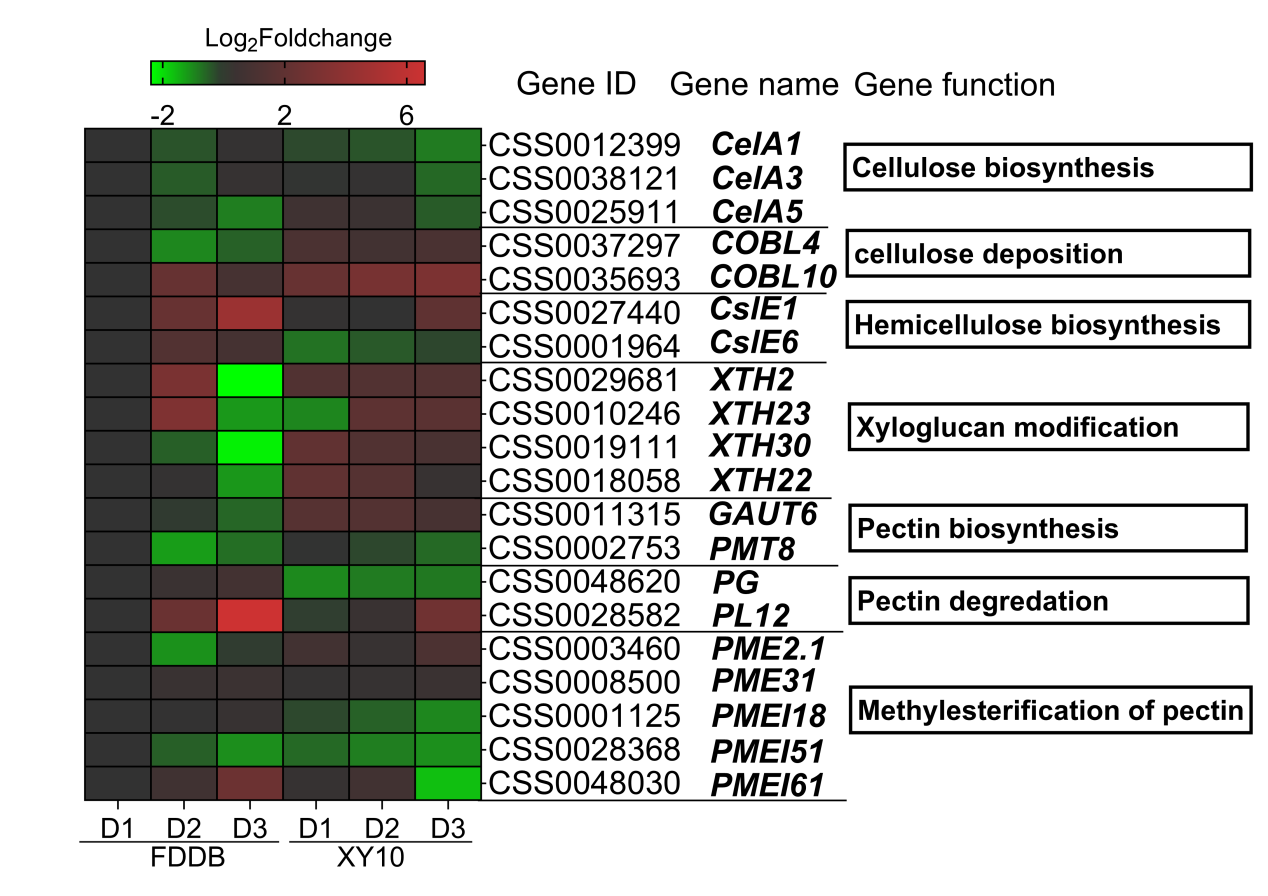


**Figure S4** Heat map of the RNA-seq data profiling of the cell wall related DEGs.

**Table S1 Summary of sample sequencing data quality**

| Sample | Raw reads | Clean reads | Clean bases | Error | Q20(%) | Q30(%) | GC(%) |
| --- | --- | --- | --- | --- | --- | --- | --- |
| D1FDDB_1 | 44,567,212 | 42,479,388 | 6.37G | 0.03 | 97.55 | 93.30 | 44.17 |
| D1FDDB_2 | 42,557,542 | 40,551,020 | 6.08G | 0.03 | 97.54 | 93.24 | 44.29 |
| D1FDDB_3 | 45,096,568 | 42,977,350 | 6.45G | 0.03 | 97.29 | 92.67 | 44.24 |
| D1XY10_1 | 41,595,066 | 40,245,636 | 6.04G | 0.03 | 97.47 | 93.07 | 44.20 |
| D1XY10_2 | 43,614,406 | 41,327,094 | 6.20G | 0.03 | 97.62 | 93.52 | 44.42 |
| D1XY10_3 | 41,290,822 | 39,386,586 | 5.91G | 0.03 | 97.67 | 93.57 | 44.04 |
| D2FDDB_1 | 44,665,172 | 43,196,336 | 6.48G | 0.03 | 97.58 | 93.30 | 44.15 |
| D2FDDB_2 | 42,349,364 | 40,540,320 | 6.08G | 0.03 | 97.38 | 92.90 | 44.00 |
| D2FDDB_3 | 41,923,030 | 40,613,370 | 6.09G | 0.03 | 97.39 | 92.87 | 44.04 |
| D2XY10_1 | 44,449,570 | 42,394,160 | 6.36G | 0.03 | 97.33 | 92.80 | 44.25 |
| D2XY10_2 | 39,760,396 | 38,094,318 | 5.71G | 0.03 | 97.15 | 92.49 | 44.30 |
| D2XY10_3 | 44,805,596 | 43,050,896 | 6.46G | 0.03 | 97.62 | 93.45 | 44.14 |
| D3FDDB_1 | 44,763,290 | 42,324,464 | 6.35G | 0.03 | 97.77 | 93.95 | 44.14 |
| D3FDDB_2 | 42,470,104 | 40,290,228 | 6.04G | 0.03 | 97.42 | 92.98 | 44.15 |
| D3FDDB_3 | 43,480,954 | 40,966,160 | 6.14G | 0.03 | 97.74 | 93.80 | 44.19 |
| D3XY10_1 | 42,856,014 | 41,573,034 | 6.24G | 0.03 | 97.43 | 92.98 | 44.25 |
| D3XY10_2 | 46,340,432 | 45,012,222 | 6.75G | 0.03 | 97.66 | 93.65 | 44.30 |
| D3XY10_3 | 44,990,946 | 41,320,894 | 6.20G | 0.03 | 95.65 | 90.40 | 41.51 |

**Table S2** GO terms for the 198 DEGs overlapped among the pairwise comparisons of consecutive sampling time points in the two varieties.

| Category | GO ID | Description | padj | Count |
| --- | --- | --- | --- | --- |
| BP | GO:0006073 | cellular glucan metabolic process | 0.0001 | 6 |
| BP | GO:0044042 | glucan metabolic process | 0.0001 | 6 |
| BP | GO:0044264 | cellular polysaccharide metabolic process | 0.0001 | 6 |
| BP | GO:0005976 | polysaccharide metabolic process | 0.0002 | 6 |
| BP | GO:0044262 | cellular carbohydrate metabolic process | 0.0006 | 6 |
| BP | GO:0009832 | plant-type cell wall biogenesis | 0.0795 | 1 |
| BP | GO:0010215 | cellulose microfibril organization | 0.0795 | 1 |
| BP | GO:0043062 | extracellular structure organization | 0.0795 | 1 |
| BP | GO:0070726 | cell wall assembly | 0.0795 | 1 |
| BP | GO:0071668 | plant-type cell wall assembly | 0.0795 | 1 |
| BP | GO:0009664 | plant-type cell wall organization | 0.0795 | 1 |
| BP | GO:0071669 | plant-type cell wall organization or biogenesis | 0.0795 | 1 |
| BP | GO:0030198 | extracellular matrix organization | 0.0795 | 1 |
| BP | GO:0045229 | external encapsulating structure organization | 0.3940 | 1 |
| BP | GO:0071555 | cell wall organization | 0.3940 | 1 |
| BP | GO:0071554 | cell wall organization or biogenesis | 0.4601 | 1 |
| BP | GO:0042546 | cell wall biogenesis | 0.0923 | 1 |
| BP | GO:0009733 | response to auxin | 0.0371 | 3 |
| BP | GO:0009719 | response to endogenous stimulus | 0.0478 | 3 |
| BP | GO:0009725 | response to hormone | 0.0478 | 3 |
| BP | GO:0010033 | response to organic substance | 0.0478 | 3 |
| BP | GO:0042221 | response to chemical | 0.0795 | 3 |
| BP | GO:0006887 | exocytosis | 0.0478 | 2 |
| BP | GO:0032940 | secretion by cell | 0.0478 | 2 |
| BP | GO:0046903 | secretion | 0.0478 | 2 |
| BP | GO:0016192 | vesicle-mediated transport | 0.0795 | 3 |
| BP | GO:0045454 | cell redox homeostasis | 0.0795 | 3 |
| BP | GO:0019725 | cellular homeostasis | 0.0795 | 3 |
| BP | GO:0042592 | homeostatic process | 0.0795 | 3 |
| BP | GO:0016567 | protein ubiquitination | 0.0795 | 2 |
| BP | GO:0032446 | protein modification by small protein conjugation | 0.0795 | 2 |
| BP | GO:0070647 | protein modification by small protein conjugation or removal | 0.1394 | 2 |
| BP | GO:0006413 | translational initiation | 0.1276 | 1 |
| BP | GO:0065008 | regulation of biological quality | 0.0892 | 3 |
| BP | GO:0007165 | signal transduction | 0.4461 | 2 |
| BP | GO:0023052 | signaling | 0.4461 | 2 |
| BP | GO:0016049 | cell growth | 0.0795 | 1 |
| BP | GO:0040007 | growth | 0.0795 | 1 |
| BP | GO:0022607 | cellular component assembly | 0.4909 | 1 |
| BP | GO:0044085 | cellular component biogenesis | 0.5961 | 1 |
| BP | GO:0016043 | cellular component organization | 0.8107 | 1 |
| CC | GO:0005576 | extracellular region | 0.0000 | 7 |
| CC | GO:0048046 | apoplast | 0.0000 | 6 |
| CC | GO:0071944 | cell periphery | 0.0000 | 8 |
| CC | GO:0005618 | cell wall | 0.0000 | 6 |
| CC | GO:0030312 | external encapsulating structure | 0.0000 | 6 |
| CC | GO:0000145 | exocyst | 0.0074 | 2 |
| CC | GO:0005938 | cell cortex | 0.0074 | 2 |
| CC | GO:0044448 | cell cortex part | 0.0074 | 2 |
| CC | GO:0099568 | cytoplasmic region | 0.0074 | 2 |
| CC | GO:0099023 | tethering complex | 0.0081 | 2 |
| CC | GO:0031225 | anchored component of membrane | 0.0548 | 1 |
| MF | GO:0016762 | xyloglucan:xyloglucosyl transferase activity | 0.0000 | 6 |
| MF | GO:0016831 | carboxy-lyase activity | 0.4296 | 1 |
| MF | GO:0046527 | glucosyltransferase activity | 0.0002 | 6 |
| MF | GO:0016830 | carbon-carbon lyase activity | 0.4386 | 1 |
| MF | GO:0005509 | calcium ion binding | 0.0002 | 8 |
| MF | GO:0043565 | sequence-specific DNA binding | 0.0096 | 6 |
| MF | GO:0015035 | protein disulfide oxidoreductase activity | 0.0233 | 3 |
| MF | GO:0015036 | disulfide oxidoreductase activity | 0.0502 | 3 |
| MF | GO:0016667 | oxidoreductase activity, acting on a sulfur group of donors | 0.0928 | 3 |
| MF | GO:0009055 | electron transfer activity | 0.2051 | 3 |
| MF | GO:0004842 | ubiquitin-protein transferase activity | 0.2202 | 2 |
| MF | GO:0019787 | ubiquitin-like protein transferase activity | 0.2202 | 2 |
| MF | GO:0003743 | translation initiation factor activity | 0.4296 | 1 |
| MF | GO:0004222 | metalloendopeptidase activity | 0.4386 | 1 |
| MF | GO:0004175 | endopeptidase activity | 0.4386 | 2 |
| MF | GO:0008237 | metallopeptidase activity | 0.4386 | 1 |
| MF | GO:0008135 | translation factor activity, RNA binding | 0.4386 | 1 |
| MF | GO:0051536 | iron-sulfur cluster binding | 0.4386 | 1 |
| MF | GO:0051540 | metal cluster binding | 0.4386 | 1 |
| MF | GO:0030170 | pyridoxal phosphate binding | 0.4537 | 1 |
| MF | GO:0070279 | vitamin B6 binding | 0.4537 | 1 |
| MF | GO:0004252 | serine-type endopeptidase activity | 0.4537 | 1 |
| MF | GO:0019842 | vitamin binding | 0.4846 | 1 |
| MF | GO:0008236 | serine-type peptidase activity | 0.6781 | 1 |
| MF | GO:0017171 | serine hydrolase activity | 0.6781 | 1 |
| MF | GO:0016829 | lyase activity | 0.7368 | 1 |
| MF | GO:0043531 | ADP binding | 0.7858 | 1 |
| MF | GO:0003723 | RNA binding | 0.8516 | 1 |

**Table S3** Primer sequences for qRT-PCR.

| Gene | Forward | Reverse |
| --- | --- | --- |
| GAPDH | TTGGCATCGTTGAGGGTCT | CAGTGGGAACACGGAAAGC |
| CelA1 | CCTCCCACCACAGTCCTTA | GATGACCCAAATGGCAAAG |
| CelA3 | GTTATTGGTGGCGTTTCGG | TCATCTGATGCCTTGGAGG |
| CelA5 | CGGAACACTGTATTCTTTG | TCTTTGGAGCATCATAACC |
| COBL4 | GGATGAACTACACGCTCTG | ATTGATGGACTGATAGGGA |
| COBL10 | CCACCAATCAGAATAGACCC | GAGAACGAGACGCAACACC |
| CslE1 | TGGCGGTACACTTCAAGGT | AGAAGGCAAAGCAATAAGA |
| CslE6 | GTTACCCTTTCAAGCACAG | ATTGTTGGAGGCTCTATTT |
| XTH2 | TATCTGGACAGCCATACAC | TAGTACACTGATGCGTTGA |
| XTH23 | GATGATTGGGCAACAAGAG | TTGGCATTGAAGTTTCTGTA |
| XTH30 | ATGAGGCAACAGTTCAAGG | ATGCTGGTCTCCATAGTTCTT |
| XTH22 | CGGAGCATCTTCTTGTAGT | TGTGGGAAACGCTTTGAGT |
| GAUT6 | AGGGATCTGACAGGACTTT | CCACCATTGGGTCTGAAAA |
| PMT8 | AAAGAAGCATTTACCAGCAT | ACAAGCACAAAGTCATCCC |
| PG | TTTGAAGACAAGCATCGGTAG | CTGGAAGAGCATTTGGGTC |
| PL | TCAATAGTCAGGGTAATCG | CGTCTGTCCTCCAGTTCCA |
| PME2.1 | AAGGACAGCCGAGTCACAT | AACCCGTCTAGGCAAGTAA |
| PME31 | ACCGTTCAGCAACACTCGC | TCTTGGTCTTGGGCACATA |
| PMEI18 | AGTTGCGTTACGTGTTGGA | CTGCGTTGCCGAAGATGAA |
| PMEI51 | GGTTCTTCATTATTCCCAGTA | CTTCAACGCAGACCAGCAA |
| PMEI61 | ACGGCTGAGGCTAGTAAGT | TATTGGAACCGATAGTATGAC |
